# Automated Detection of Carotid Artery Stenosis Using a Sensitive Accelerometer Wearable Sensor and Interpretable Machine Learning

**DOI:** 10.3390/bios16050238

**Published:** 2026-04-23

**Authors:** Houriyeh Majditehran, Brian Sang, Nia Desai, Fadi Nahab, Nino Kvantaliani, Debra Blanke, Danielle Starnes, Hannah Christopher, Jin-Woo Park, Farrokh Ayazi

**Affiliations:** 1 School of Electrical and Computer Engineering, Georgia Institute of Technology, Atlanta, GA 30332, USA; 2 Department of Medicine, Emory University School of Medicine, Atlanta, GA 30322, USA; 3 StethX Microsystems Inc., Atlanta, GA 30308, USA

**Keywords:** wearable biosensor, stenosis, MEMS accelerometer, feature extraction, explainable machine learning, seismic patch, carotid artery disease

## Abstract

Carotid artery disease, including atherosclerotic stenosis and non-atherosclerotic abnormalities, substantially increases ischemic stroke risk and motivates accessible tools for early screening. Current diagnostic pathways rely on clinic-based imaging and skilled operators, creating barriers to frequent monitoring and scalable deployment. We present a non-invasive diagnostic approach using a wearable MEMS accelerometer patch to capture mechano-acoustic vibrations generated by carotid blood flow at the neck. The miniature device integrates a hermetically sealed wideband accelerometer with out-of-plane sensitivity and micro-g resolution to detect subtle flow-induced vibrations. We validated the approach in a carotid flow phantom and a clinical study of 74 patients. Time–frequency representations were computed using the continuous wavelet transform (CWT), from which interpretable spectral and scalogram-derived candidate biomarkers were extracted. Six non-redundant features were then selected for multivariate classification, distinguishing pathology, defined as 50% or greater stenosis or a non-atherosclerotic abnormality, from non-pathology, defined as less than 50% stenosis. Finally, model interpretability was assessed using SHapley Additive exPlanations (SHAP) to quantify the contribution of each biomarker to predicted disease probability. These findings resulted in an AUROC of 0.97 and AUPR of 0.947, with 81.7% sensitivity and 93.6% specificity at the prespecified threshold (precision 85.4%, F1 83.5%, accuracy 89.8%), highlighting the potential of wearable seismic sensing combined with interpretable machine learning for fast screening and longitudinal monitoring of the right and left carotid arteries.

## 1. Introduction

**Figure 1:**
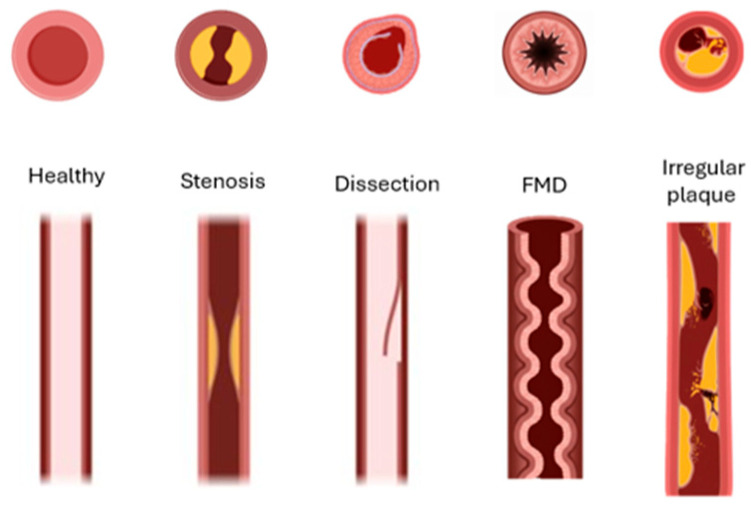
Schematic depictions of (left to right) normal artery, atherosclerotic stenosis, dissection, fibromuscular dysplasia (FMD), irregular/ulcerated atherosclerotic plaque, and combined dissection.

**Figure 2:**
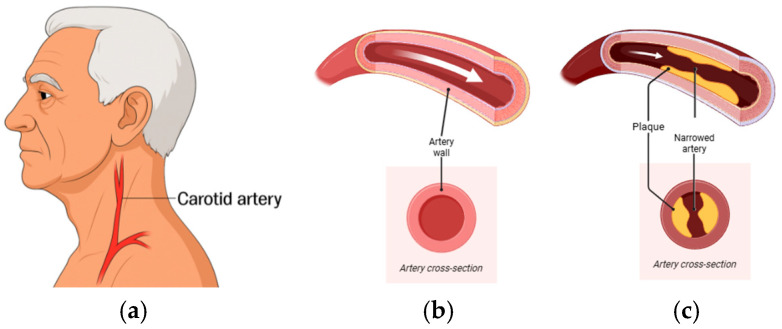
(**a**) Location of the carotid artery in the neck. (**b**) Healthy artery showing an open lumen and normal blood flow. (**c**) Diseased artery with atherosclerotic plaque accumulation, leading to narrowing of the lumen and reduced cross-sectional area for flow and expected hemodynamic consequences. White arrows indicate the direction of blood flow.

Stroke is a leading global cause of death and long-term disability, responsible for over seven million deaths annually worldwide [1]. Up to 20% of ischemic strokes are attributed to carotid artery (CA) disease, principally atherosclerotic stenosis but also vessel dissection and other structural abnormalities [2]. Because the majority of CA disease is initially asymptomatic, early detection is essential to prevent cerebrovascular events. Despite advances in vascular imaging, frontline tools—including duplex ultrasound (DUS), computed tomography angiography (CTA), and magnetic resonance angiography (MRA)—have practical constraints for broad deployment. DUS is widely used but remains operator-dependent and shows variable accuracy across stenosis strata; recent reviews advise caution when using DUS alone for preoperative decision-making [3,4,5]. CTA and MRA improve anatomic characterization but require specialized infrastructure, expose patients to radiation or contrast (CTA), or are susceptible to motion and availability constraints (MRA); contemporary stroke or transient ischemic attack (TIA) guidelines recommend using MRA or CTA to confirm ultrasound-detected ≥50% stenosis before management decisions [6,7]. Beyond imaging, carotid bruit auscultation provides only modest diagnostic performance and is inadequate as a stand-alone screening test [8]. When disease is suspected or detected using auscultation or ultrasound, contemporary guidelines advise confirmation with CTA or MRA before management decisions, underscoring the need for accessible, quantitative, hemodynamics-based methods to triage patients for confirmatory imaging [9,10]. Delayed diagnosis of clinically significant carotid stenosis has major consequences. Patients presenting with TIA or minor stroke face a high early risk of recurrent events, estimated at 8–12% within the first seven days and up to 20% by 90 days if untreated [11]. Moreover, carotid endarterectomy (CEA) and stenting substantially reduce this risk, but the magnitude of benefit is highly time-dependent. Surgical intervention within fourteen days of symptoms yields the greatest stroke-prevention effect, whereas postponement beyond this window markedly diminishes efficacy. Indeed, pooled analyses of landmark trials show that when revascularization is performed within two weeks, the number needed to treat (NNT) to prevent one stroke is approximately 5, but if delayed beyond 12 weeks, the NNT rises dramatically to about 125. This compressed therapeutic window underscores the urgency of timely detection and referral highlighting the limitations of current pathways that rely on access to specialized imaging and vascular services [12]. The carotid lesion variants are shown in Figure 1. Atherosclerotic stenosis produces fixed luminal narrowing; dissection reflects an intimal tear with an intimal flap and potential false lumen; fibromuscular dysplasia (FMD) is a non-atherosclerotic arteriopathy with a multifocal “string-of-beads” pattern; and irregular/ulcerated atherosclerotic plaque indicates surface disruption. Each morphology alters local flow and wall motion in ways expected to imprint distinct acoustic signatures on carotid vibrations Figure 2. Flow disturbances from luminal narrowing or wall injury generate low-amplitude mechano-acoustic vibrations that can be detected at the skin surface.

Recent studies have shown that a low-profile wearable patch using a wideband sensitive MEMS accelerometer can capture physiologic mechano-acoustic signatures with high fidelity in hospital settings and support machine learning-based classification of pathology in related cardiopulmonary tasks [13,14] and carotid artery assessments [15]. Building on this premise, we evaluate the performance of a small wearable seismic device that utilizes a sensitive accelerometer coupled with time–frequency analysis and machine learning to characterize carotid pathology in a clinical study. While stethoscope-based auscultation of carotid bruits shows limited diagnostic performance (high specificity but modest sensitivity), advanced vibration sensing and signal processing may recover richer, disease-relevant information [8]. Time–frequency analysis is well suited to such nonstationary biosignals. The seismocardiography literature demonstrates that continuous wavelet transforms (CWTs) and related time–frequency distributions capture morphology and timing of physiologic micro-vibrations linked to cardiac and vascular events [16,17]. Methodologically, the wavelet framework provides principled control of time–frequency trade-offs and significance testing of wavelet power spectra, supporting reproducible biomarker extraction. In cardiopulmonary acoustics, convolutional neural networks trained on spectro-temporal features have achieved strong performance for heart- and lung-sound tasks, and residual networks (e.g., ResNet-18) offer an efficient backbone for such image-like inputs [18,19]. Rather than rely solely on deep features, we begin with hand-crafted descriptors on CWT scalograms that capture pathology-driven high-frequency shifts and time–frequency complexity. This feature-based strategy was chosen to prioritize interpretability, reduce overfitting risk in a limited cohort, and limit computational complexity. Building on these advances, we hypothesize that carotid pathology produces characteristic shifts in vibration energy toward mid-to-higher frequencies and that learned representations of wavelet scalograms can distinguish carotids more likely to require further clinical attention from lower-stenosis carotids with high accuracy. This study presents a lightweight wearable MEMS accelerometer patch for non-invasive carotid vibration sensing, supported by phantom experiments, prospectively collected clinical recordings, and interpretable machine learning analysis for automated carotid screening. To our knowledge, this is the first full-length clinical study of a lightweight wearable accelerometer patch for automated, non-invasive carotid stenosis screening.

## 2. Materials and Methods

**Figure 3:**
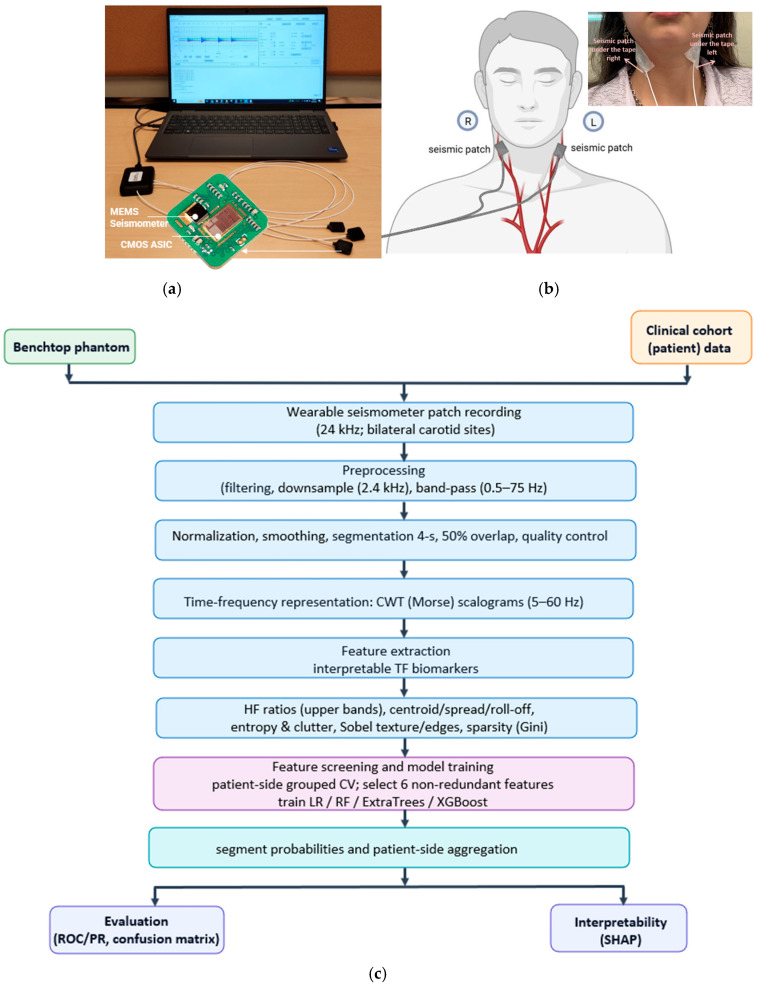
(**a**): The StethX seismic patch incorporates a hermetically sealed MEMS seismometer with normal-axis (out-of-plane) sensitivity. The sensor integrates the MEMS die and its CMOS ASIC in a single sealed package. (**b**): Sensor placement during patient data collection on right and left carotid artery areas. (**c**): Phantom and clinical data are processed via bilateral patch recordings, preprocessing and 4 s segmentation, CWT (Morse) scalograms (5–60 Hz), interpretable feature extraction, patient-side grouped CV model training (LR/RF/ExtraTrees/XGBoost), segment-to-patient aggregation, and evaluation (ROC/PR, confusion matrix) with SHAP interpretability.

An overview of the wearable seismic patch, neck placement, and end-to-end analysis pipeline is shown in Figure 3. The wearable seismic patch (StethX, Atlanta, GA, USA) uses a hermetically sealed, wideband micro-g MEMS accelerometer with high out-of-plane sensitivity to capture low-amplitude, flow-induced neck vibrations, with reduced susceptibility to airborne acoustic noise in clinical environments. For both phantom and clinical recordings, the patch was secured to the skin using medical-grade 3M 2764 adhesive tape (3M Company, St. Paul, MN 55144-1000, USA) to promote consistent mechanical coupling. Figure 3a also summarizes the acquisition setup, including the StethX patch array connected to a multichannel interface and laptop-based recording software. Detailed device specifications and noise characterization are provided in the Supplementary Materials (Figure S1).

### 2.1. Study Design and Setting

To evaluate the feasibility of a wearable seismic patch for non-invasive assessment of carotid artery disease, we conducted a prospective, single-center diagnostic study at the Emory Stroke Clinic (Atlanta, GA, USA). The study protocol was approved by the Emory University and Georgia Institute of Technology Institutional Review Board (IRB #00105563). Written informed consent was obtained from all participants. All experiments were performed in accordance with relevant guidelines and regulations. Enrollment focused on patients with imaging-confirmed carotid artery disease, including stenosis, cervical artery dissection, FMD, and irregular or ulcerative atherosclerotic plaque. Because these conditions are relatively low-incidence, recruitment required more than one year of continuous data collection by the research team. Onsite collection ensured standardized sensor placement and high-quality recordings, though this approach also limited the overall number of participants and led to class imbalance across disease subgroups.

### 2.2. Participants

A total of 74 patients were prospectively recruited. Eligible participants had a previously confirmed carotid artery condition—stenosis or dissection—diagnosed within the prior year by computed tomography angiography (CTA), magnetic resonance angiography (MRA), or carotid ultrasound. Patients represented a spectrum of ages, sexes, and stenosis severities, with demographics and clinical details summarized in Supplementary Table S1. Recruitment and data collection were performed onsite by the research team over a period exceeding one year. Because participants were recruited from a specialty stroke clinic population undergoing evaluation for carotid disease, this pilot cohort did not include a separate lesion-free healthy-control group. After prespecified exclusions, such as inadequate recording quality, or recordings contaminated by coughing or swallowing, 71 patients (mean age 63.44 ± 13.99 years; 31 male, 40 female; BMI 26.97 ± 5.15 kg/m^2^) remained for analysis.

### 2.3. Imaging Modalities and Clinical Labels

**Figure 4:**
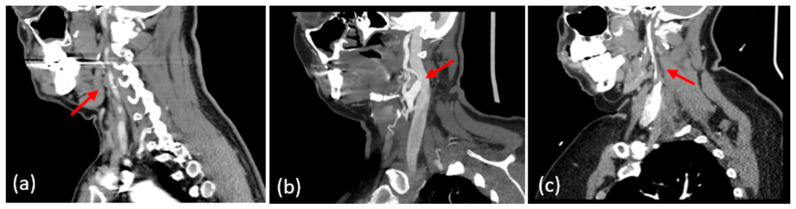
Representative CTA phenotypes. (**a**) Carotid atherosclerotic stenosis with focal luminal narrowing (red arrow). (**b**) Carotid artery dissection with tapered lumen and intimal flap (red arrow). (**c**) Fibromuscular dysplasia showing multifocal “string-of-beads” narrowing of the distal cervical ICA (red arrow).

Ground-truth labels for carotid pathology were assigned based on the available reference imaging for each side of the neck. CTA was used when present to provide a high-resolution assessment of luminal narrowing, plaque morphology, and vessel-wall features. MRA/MRI, including vessel-wall sequences when available, was used to identify dissections and intramural hematomas. Duplex ultrasound was used to quantify stenosis by applying SRU/IAC Doppler velocity thresholds and ICA/CCA ratios when cross-sectional angiography was not available. Representative examples of each phenotype on CTA are shown in Figure 4. Panel (a) demonstrates focal carotid atherosclerotic stenosis with a severe luminal narrowing (arrow). Panel (b) shows a carotid dissection with a tapered lumen and intimal flap (arrow). Panel (c) depicts fibromuscular dysplasia with multifocal “string-of-beads” narrowing in the distal cervical ICA (arrow). These examples illustrate the imaging features used for label assignments in our dataset. For this study, patients were grouped into <50% stenosis and ≥50% stenosis categories. This binary stratification was selected to distinguish patients below versus at a clinically meaningful stenosis threshold, and to maintain sufficient sample size per class for reliable model development in this pilot cohort. This grouping is based on clinical actionability, rather than the absence of disease. The goal of this study was to identify the subgroup of patients in whom clinical management may change rather than distinguishing completely healthy arteries from mild disease. As such, the <50% group should not be interpreted as a healthy control population, but rather as a lower-stenosis clinical group where no clinical action is taken. Because the present dataset was collected from a clinic-based population with previously identified carotid abnormalities, it did not include a separate lesion-free healthy-control cohort. In addition, more granular stratification by stenosis severity or by pathology subtype was not pursued in the current study because several subgroups would have contained too few samples for statistically reliable modeling. These questions are important and will be addressed in future larger studies with expanded cohort sizes and broader clinical characterization.

### 2.4. Data Processing

#### 2.4.1. Signal Preprocessing

Raw vibration signals from the right and left carotid arteries (RCA and LCA) were acquired at a 24 kHz sampling rate, converted to double precision with wrap-around handling, and downsampled by a factor of 10 to 2.4 kHz for subsequent analysis. Each participant underwent two consecutive 1 min recordings while seated upright and instructed to remain still and avoid speaking. The sensor was first placed on the upper neck and then repositioned slightly lower on the neck for the second recording. The second placement was included to assess sensitivity to small variations in sensor location; preliminary analysis showed no significant difference between positions, so recordings from both positions were used for downstream analysis. Each channel was processed using a fourth-order Butterworth band-pass filter with cutoff frequencies of 0.5 and 75 Hz (−3 dB) [20], followed by per-channel z-score normalization. To reduce residual jitter while preserving overall waveform morphology, Savitzky–Golay smoothing and a centered moving-average filter were then applied [21]. The first 2 s of each recording were discarded to remove initialization transients, and all channels were trimmed to a common length before segmentation. Finally, each RCA and LCA signal was divided into 4 s windows with a 2 s hop (50% overlap), and only complete segments were retained for downstream analysis.

#### 2.4.2. Time–Frequency Representation or Continuous Wavelet Transform Representation

**Figure 5:**
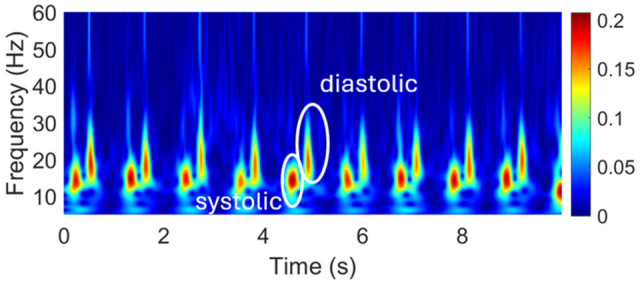
Continuous wavelet transform (CWT) of a representative carotid vibration signal, showing the time–frequency distribution of vibrational energy in the 5–60 Hz band. Peaks in energy correspond to physiological events within each cardiac cycle.

For each segment, we computed a continuous wavelet transform (CWT) to localize frequency information with the default analytic Morse wavelet. We used the magnitude of the complex coefficients as the time–frequency representation. For a preprocessed segment x(t), the CWT is defined as

$$W\left(a,b\right)=\frac{1}{\sqrt{a}}\int_{-\infty }^{\infty }x\left(t\right)\;\psi^{*}\;\left(\frac{t-b}{a}\right)\;dt$$

where a denotes scale, b  denotes time shift, and ψ is the analytic Morse wavelet. The magnitude of the complex coefficients, ∣W(a,b)∣, was used as the time–frequency representation for subsequent analysis. Analysis was restricted to the 5–60 Hz band, which contained the dominant carotid vibration content after preprocessing. This scalogram representation served as the input for downstream feature extraction. A representative CWT scalogram is shown in Figure 5. For each segment, the CWT was computed using the analytic Morse wavelet. MATLAB R2024a (MathWorks, Natick, MA, USA)’s default CWT implementation uses the analytic Morse $\left(3,60\right)$ wavelet, where 3 is the symmetry parameter and 60 is the time–bandwidth product. We used this standard setting throughout the study because it provides a suitable balance between time and frequency localization for nonstationary vibration signals; in particular, γ=3 is recommended for CWT analysis, and larger time–bandwidth values improve frequency localization at the expense of broader temporal support. A visualization of the wavelet and its filter-bank frequency responses is provided in Supplementary Figure S4.

### 2.5. Feature Extraction

Hand-crafted features were extracted from time–frequency (TF) representations. Prior biomedical acoustics consistently reports two interpretable TF feature families: (i) band-energy ratios that quantify pathology-driven upward spectral shifts, and (ii) entropy measures that summarize TF complexity. In carotid bruits, spectral analyses show that increasing stenosis severity is associated with a greater proportion of high-frequency energy, including recent work using electronic-stethoscope recordings, motivating our use of the ratio of high-frequency (HF) to total and HF ratio to low power ratios as interpretable markers [22,23]. Mechanistically, stenosis can create a high-velocity jet with downstream flow turbulence; the resulting rapid pressure and velocity fluctuations can excite higher-frequency vessel-wall vibrations, increasing the relative high-frequency content of the measured carotid vibration signal. In vascular Doppler, power–frequency spectrum analysis explicitly measured energy in selected higher-frequency bands to separate diseased from control arteries, again motivating HF-weighted band power ratios [24]. In heart sounds, wavelet-domain studies often compute sub-band entropies (Shannon/Rényi/Tsallis) and report strong discrimination for murmurs; multiple reports identify Rényi and Shannon entropies as among the most informative sub-band features [25,26,27]. Related PCG work further shows that HPSS-derived spectral descriptors—centroid, bandwidth, and 85% roll-off—along with RMS/MFCC/chroma features, can be effective for heart-sound classification, reinforcing the value of energy-location descriptors [28]. In lung sounds, combining DWT (Discrete Wavelet Transform) or WPD (Wavelet Packet Decomposition) band energies with entropy measures yields high accuracy, with WPD and Rényi entropy frequently outperforming other entropy variants [29,30]. Reviews in seismocardiography similarly emphasize compact band power and entropy features within physiologic bands as effective and interpretable descriptors [16]. Beyond biomedicine, seismic-signal classification using CWT scalograms paired with image-space energy features (e.g., band-limited intensity sums/ratios) and wavelet-based descriptors has also demonstrated the effectiveness of scalogram-plus hand-crafted pipelines [31]. Guided by this literature, we compute from CWT scalograms high-frequency power proportions and entropy features on the full map and HF-restricted stripes to be especially informative in line with prior findings. Accordingly, for each 4 s segment we used the wavelet time–frequency representation (CWT; analysis band 5–60 Hz) chosen after preliminary inspection of a wider band suggested limited additional structure above 60 Hz; this range is also consistent with common mechanocardiography preprocessing in BCG and SCG studies and reports that carotid vascular-sound energy is concentrated mainly in the tens of hertz [32,33,34]. We then derive a compact, interpretable feature set that captures four aspects of the signal: how much energy lies at higher frequencies, at what frequencies the energy is concentrated, how dispersed or cluttered the pattern is, and how sharp the time–frequency texture appears. High-frequency content was quantified by the proportion of energy in the upper half of the analysis band (high-frequency proportion) and by high-vs-low energy ratios defined over upper fractional bands (upper 30/40/50% relative to the complementary band and to the total). Energy location was summarized by the spectral centroid and spread along the frequency axis and by roll-off positions at 85% and 95% cumulative energy. Clutter/dispersion was measured using spectral-image entropy computed on the full band and as upper-to-lower band ratios. Texture/edges were captured by the Sobel gradient magnitude of the scalogram, reported globally and as an upper-to-lower band ratio. All features were computed from the magnitude of the CWT and normalized to be scale-invariant; precise definitions and parameters are provided in Supplementary Table S2. To justify this methodology, we note that band-energy ratios are widely used in cardio-respiratory acoustics. In respiratory sound analysis, investigators routinely compute power within predefined bands (e.g., 100–200 Hz, 200–400 Hz) and compare bands or band-to-total energy to characterize pathology and wheeze content [35,36,37]. In seismocardiograms, normalized band-energy-to-total-energy ratios across the physiologic band (0.5–50 Hz) are used to study posture and inter-recording variability, illustrating the same normalization principle we adopt [38]. For carotid bruits, classical spectral studies show that increasing stenosis shifts the energy toward higher frequencies (higher peak/break frequencies and a greater proportion of high-frequency components), motivating features that emphasize the upper portion of the analysis band [22,39]. To quantify high-frequency content we used upper-band/total and upper-band/lower-band energy ratios, consistent with prior work in heart sounds (band-passed-to-total energy), seismocardiography (band-to-total and subaudible-to-audible ratios), and lung sounds (high-band power ratios for wheeze detection) [19,40,41,42]. Prior work typically defines high-frequency ratios on the power spectrum using fixed bands (e.g., band-to-total or band-to-band ratios [40,41,42,43]. We adopt the same idea but compute energy directly from the wavelet time–frequency representation, using a fractional upper band (e.g., upper 50% of 5–60 Hz about 32.5–60 Hz) to form upper-band/total and upper-band/lower-band ratios. Band-energy ratios were computed from the CWT energy rather than the PSD: for a chosen analysis band (5, 60) Hz, we integrate ${\left|W_{x}\left(f_{,}t\right)\right|}^{2}$ over the upper fraction of the band (for instance, upper 50% = 32.5–60 Hz) and normalize by either the total band energy or the complementary lower fraction.

### 2.6. Classifier, Cross-Validation and Feature Ranking Procedure

For feature ranking, a single baseline classifier (the model used in the main analysis) was evaluated under subject-grouped cross-validation to prevent leakage across folds. The dataset included 71 patients and 128 carotid sides. Out of 71 patients, 29 had at least one abnormal side; at the side level, 39 out of 128 sides were abnormal. For each feature considered in isolation, the classifier was trained on the training folds and evaluated on the validation folds using area under the ROC curve (AUC), F1-score, precision–recall AUC (PR-AUC), and accuracy. Performance values were averaged across folds. Hyperparameters were held fixed across features to ensure a fair comparison and to minimize feature-specific tuning effects. Within each metric, features were ranked, with the best score assigned rank 1 (higher-is-better). A mean rank was then obtained for each feature by averaging its ranks across AUC, F1, PR-AUC, and accuracy, yielding a single comparative score in which lower values indicate stronger overall discriminative power.

#### 2.6.1. Univariate Feature Analysis

To assess which features were individually most discriminative, we performed a univariate supervised analysis on the training set, similar to previous radiomics studies that screen imaging features using cross-validated ROC analysis [44,45]. Using 5-fold stratified group cross-validation with carotid-side as the grouping variable, we treated each candidate feature as a continuous score for predicting stenosis and computed its area under the ROC curve (AUROC) and area under the precision–recall curve (AUPR) on held-out folds. For each metric, features were ranked in descending order of performance; we then averaged the AUROC and AUPR ranks to obtain a single mean rank per feature. A compact set of highly ranked, physiologically interpretable and non-redundant features was then selected for multivariate modeling. To further characterize the final six-feature representation, we performed supplementary cumulative subset and leave-one-feature-out analyses using logistic regression under the same patient-side grouped cross-validation framework.

#### 2.6.2. Model Development and Evaluation

Traditional machine learning classifiers were trained on hand-engineered time–frequency biomarkers extracted from carotid vibration recordings. Because multiple segments were obtained from each carotid side, we used a group-aware design to prevent leakage from repeated measurements. Each segment was assigned a right or left group identifier including patient ID and laterality, and all data splitting and cross-validation were performed at the patient-side level so that segments from the same carotid side never appeared in both training and evaluation sets. We created a fixed held-out test set comprising 15% of patient-side groups selected by stratified sampling to preserve group-level class prevalence. All remaining patient-side groups formed the training set. Models were fit using the training set only and evaluated once on the held-out test set. We evaluated L2-regularized logistic regression, random forest, ExtraTrees, and XGBoost classifiers. These models were selected because the study focused on a compact set of interpretable vibration-derived biomarkers in a small pilot cohort with grouped evaluation by carotid side, where feature-based traditional machine learning models are less prone to overfitting and more clinically interpretable than higher-capacity deep learning approaches. For logistic regression, continuous features were standardized using statistics computed on the training data. Class imbalance was addressed using class weights for the linear model and balanced sampling/weights for tree-based models. A compact multivariate feature set of six biomarkers was used for multivariate modeling to reduce redundancy while preserving interpretability. The six-feature set was retained as a compact, interpretable, and non-redundant subset capturing complementary signal characteristics. Supplementary leave-one-feature-out ablation used to further characterize this choice is reported in Supplementary Table S5. Demographic ablation was evaluated separately as a confounding check (Supplementary Table S4). Models produced probabilistic predictions at the segment level. Because clinical decisions are made per carotid side, we additionally computed patient-side predictions by averaging segment probabilities within each patient-side group (primary endpoint). We report performance at both resolutions: segment-level results characterize discriminative information in short time windows, whereas patient-side results reflect the clinically relevant decision unit and avoid overcounting correlated segments. Threshold-free discrimination was quantified using receiver operating characteristic and precision–recall analyses. To report thresholded metrics, we prespecified an operating threshold using out-of-fold, cross-validated patient-side predictions from the training set only. We selected the lowest threshold achieving sensitivity ≥0.90 and, among those, the one maximizing specificity, then applied it unchanged to the held-out test set for both patient-side and segment-level metrics. Model development used stratified, grouped 5-fold cross-validation at the patient-side level, and the test set was not used for preprocessing, fitting, thresholding, or model selection.

#### 2.6.3. Model Interpretability (SHAP)

SHapley Additive exPlanations (SHAP) is widely used to interpret clinical prediction models by assigning additive feature attributions to individual predictions based on Shapley values from cooperative game theory [46]. SHAP-style interpretability has also been reported for physiologic signal modeling, for instance, ECG, including analyses that relate feature attributions to time–frequency descriptors [47]. Accordingly, we computed SHAP values for the final model to quantify how each time–frequency biomarker increases or decreases the predicted probability of stenosis relative to a baseline expectation. To avoid using held-out test data for interpretation, SHAP analysis was performed on the training set after fitting the final model.

## 3. Results

### 3.1. Clinical Results

**Figure 6:**
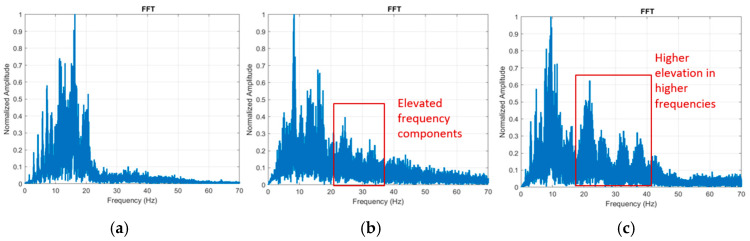
Frequency-domain spectra (FFTs) from three representative carotid-side examples (*n* = 1 carotid side per panel; three examples shown). (**a**) Representative lower-stenosis carotid example: Power concentrated <20 Hz. (**b**) LCA with >50% stenosis: Elevated 20–30 Hz components. (**c**) LCA with dissection: Stronger, broader elevation into 25–40 Hz. Axes: Normalized amplitude (y) and frequency (Hz, x; 0–70 Hz). These spectra corroborate the time–frequency patterns and illustrate pathology-dependent shifts toward higher frequencies.

Representative frequency-domain spectra mirrored the phantom dose–response. (presented in Supplementary Figure S2) In a lower-stenosis carotid patient, energy was concentrated below ~20 Hz (left panel; see healthy scalogram in Figure 6). In a patient with imaging-labeled ≥50% LCA stenosis, the FFT amplitude showed a clear elevation in the 20–30 Hz band relative to the other side (middle panel; consistent with the paired scalogram/FFT described in the text). Here, ≥50% refers to luminal carotid stenosis severity (percent diameter reduction) from the reference clinical imaging used for labeling. This cutoff is clinically used—particularly in symptomatic carotid disease—because management recommendations may change at ≥50% stenosis, including consideration of carotid endarterectomy or stenting in addition to optimal medical therapy depending on symptoms and patient risk [48,49].

### 3.2. Feature Importance and Interpretability of Time–Frequency Biomarkers

#### 3.2.1. Univariate Discriminative Ranking of Time–Frequency Biomarkers

To guide the selection of a compact and interpretable multivariate feature set, we performed univariate screening on the training set using patient-side grouped stratified 5-fold cross-validation. Each feature was evaluated in isolation using AUROC and AUPR, evaluating both score orientations (feature and its negation) and retaining the better cross-validated value for each metric. Features were ranked by the mean of their AUROC and AUPR ranks. Full rankings and additional details are provided in Supplementary Table S2 and Figure S3. As a secondary consistency check, we compared feature distributions between classes on the training set using a two-sided Mann–Whitney U test with Benjamini–Hochberg false-discovery rate (FDR) correction; to avoid non-independence from repeated segments, feature values were first aggregated at the patient-side level prior to testing (Supplementary Table S3).

#### 3.2.2. Model Selection and Performance

**Figure 7:**
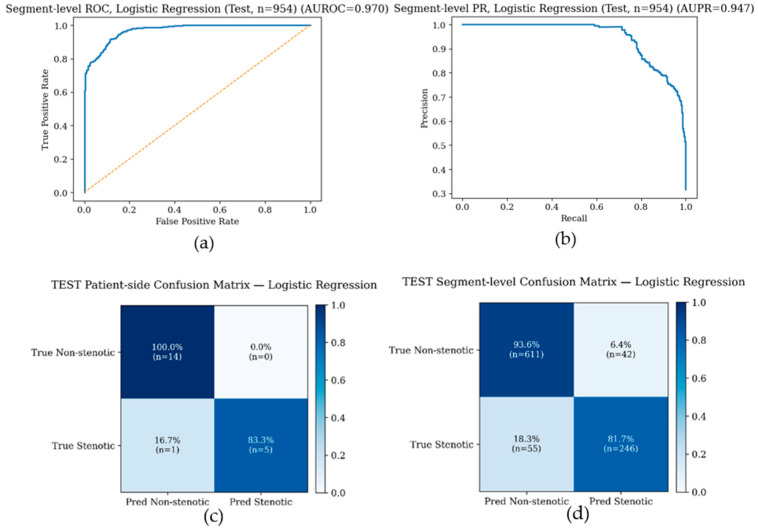
Test-set performance of the representative logistic regression model. (**a**) ROC curve and (**b**) precision–recall curve for segment-level classification with 954 segments. In panel (**a**), the solid blue line denotes the logistic regression ROC curve and the dashed diagonal line denotes the no-discrimination reference. In panel (**b**), the solid blue line denotes the precision–recall curve. (**c**) Patient-side confusion matrix at the prespecified operating threshold with total of 20 carotid sides. (**d**) Segment-level confusion matrix at the prespecified operating threshold with 954 segments. Confusion matrix entries show percentages and raw counts.

Patient-side performance was treated as the primary endpoint, because clinical decisions are made per carotid side rather than per individual segment. Segment-level results are additionally reported to assess whether discriminative information is present in short time windows. On the held-out test set, all evaluated models performed well (Table 1). We report logistic regression as a representative model: at the segment level it achieved AUROC = 0.970 and AUPR = 0.947 with sensitivity 0.817, specificity 0.936, and accuracy 0.898. As a simple benchmark, a single-feature baseline using the high-frequency power ratio of the top 50% band alone showed strong discrimination under the same patient-side grouped evaluation, consistent with the univariate ranking results. ROC and precision–recall curves for logistic regression are shown both at the segment level and after patient-side aggregation, with improved discrimination after aggregation consistent with reduced within-subject variability. At the prespecified operating threshold (selected from training predictions and applied unchanged to the test set), confusion matrices for patient-side and segment-level classification are shown in Figure 7a–d, demonstrating high specificity with few false positives. Supplementary demographic ablation analyses further showed that age, sex, height, weight, and BMI alone provided limited discrimination, and that adding these variables to the six vibration-derived features did not improve performance, suggesting that the observed classification performance was primarily driven by the vibration-derived biomarkers (Supplementary Table S4).

### 3.3. Interpretability of Time–Frequency Biomarkers

**Figure 8:**
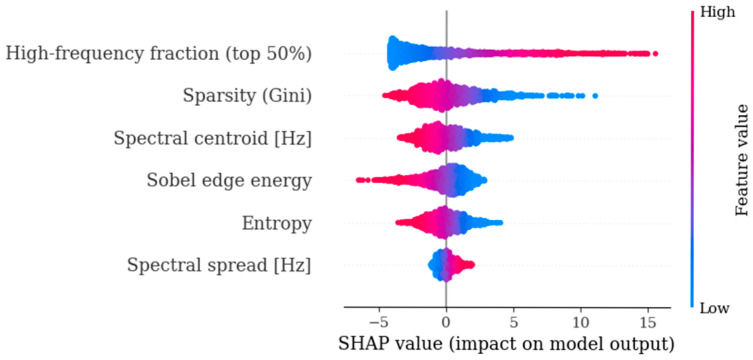
SHAP summary plot for the final logistic regression model using six time–frequency biomarkers (training set, segment level). Each point represents one segment; SHAP values indicate the contribution of each biomarker to the model output. Blue indicates lower feature values and red indicates higher feature values; the horizontal position shows how each segment shifts the prediction, and a wider spread indicates greater feature impact. Spectral centroid and spectral spread are reported in Hz; high-frequency fraction, sparsity (Gini), and entropy are unitless normalized descriptors; Sobel edge energy is reported in normalized units.

To interpret the learned decision rule, we computed SHAP values for the final logistic regression model on the training data. The SHAP summary (Figure 8) indicates that the high-frequency fraction (top 50%) is the most influential biomarker, with larger values generally increasing the predicted probability of stenosis. Sparsity (Gini) and the spectral centroid also contributed strongly, while Sobel edge energy and entropy provided complementary discriminative information. Spectral spread had the smallest effect among the selected features. Therefore, SHAP analysis suggests that the model relies primarily on high-frequency content and signal structure (sparsity) to distinguish stenotic from non-stenotic segments. As outlined in Figure 8, patient-side grouped evaluation and SHAP analysis were used to quantify discrimination performance and identify the most influential biomarkers.

**Figure 9:**
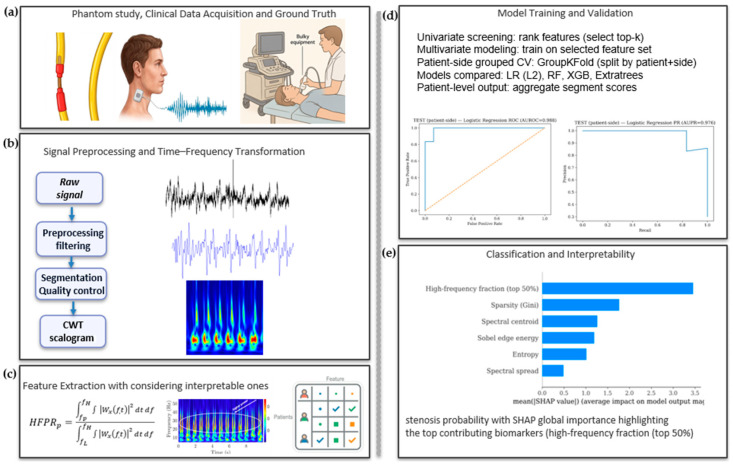
End-to-end workflow for wearable carotid stenosis screening. (**a**) Phantom/clinical acquisition with imaging ground truth. (**b**) Preprocessing, segmentation, and CWT scalogram generation (5–60 Hz). (**c**) Interpretable feature extraction. (**d**) Patient-side grouped cross-validation (GroupKFold; split by patient + side) comparing LR (L2), RF, XGB, and ExtraTrees with segment-to-patient aggregation. (**e**) Patient-side prediction with SHAP global feature importance.

Overall, our end-to-end workflow, from acquisition and preprocessing through model training and interpretability, is summarized in Figure 9.

## 4. Discussions

For the majority of the cohort, two separate recordings were obtained: the first with the seismic patch positioned on the upper neck, and the second at a lower position. Preliminary analysis revealed no statistically significant differences between the signals from these two locations, indicating that the sensor is robust to minor variations in positioning. Consequently, the data from both recording positions were pooled for the final analysis. In related work using an electronic stethoscope with spectral features and logistic regression, [23] reported a testing AUC of 0.79 (sensitivity 78.6%, specificity 72.1%) for detecting >70% carotid stenosis from distal recordings. Compared to that report, our wearable seismic patch approach achieved higher discrimination in our cohort (AUROC 0.97), although direct comparisons are limited by differences in sensing modality, cohort, and label definitions. The present study was designed as an initial clinically oriented screening analysis centered on the distinction between <50% and ≥50% carotid pathology. This grouping was chosen to align with a clinically meaningful management threshold. In this context, the <50% group should be interpreted as a lower-stenosis clinical comparator rather than a lesion-free healthy-control cohort. At the same time, this binary labeling approach does not capture the full spectrum of disease severity. For example, in our cohort, a carotid side with 50–69% stenosis showed a smaller elevation in high-frequency power ratio than cases of near-complete occlusion. This is physiologically reasonable, since moderate and near-occlusive stenoses produce different levels of jet formation, flow acceleration, and downstream disturbance, which are expected to generate different vibration signatures. Therefore, combining these severities into a single pathology class likely introduces within-class heterogeneity and may reduce the discriminative power of the model. Our findings are consistent with computational fluid dynamics studies demonstrating severity-dependent hemodynamic differences in carotid stenosis [12], supporting the use of more granular severity-based labels in future larger studies. Recent Natural Language Processing (NLP) work further shows that stenosis severity can and should be extracted from routine clinical text, enabling finer-grained labels [50]. Another important point is that, in patients with bilateral carotid disease, the functional impact of stenosis on one side may influence hemodynamic readings on the opposite side; for example, duplex ultrasound velocities often decrease in the contralateral artery after revascularization of the ipsilateral side [51]. We observed that when one carotid artery was labeled as severely stenosed, the contralateral side often exhibited subtle alterations in high-frequency features. Severe disease on the contralateral side elevates measured velocities and can lead to overreading ipsilateral stenosis [52]. Because cervical artery dissection is relatively uncommon in routine clinic flow, it comprised the smallest subgroup in our dataset [53,54]. Supplementary demographic ablation analyses further showed that age, sex, height, weight, and BMI alone provided limited discrimination, and that adding these variables to the vibration-derived features did not improve performance, suggesting that the present model is driven primarily by the vibration-derived biomarkers rather than by the available demographic variables. Future studies with larger cohorts should extend this framework to include finer severity stratification.

## 5. Conclusions

We demonstrated that wearable seismic patches using a sensitive accelerometer combined with time–frequency analysis can non-invasively capture carotid flow-induced vibrations and differentiate carotid conditions with <50% stenosis from pathologic carotid conditions. In benchtop phantom experiments, graded constriction produced a consistent frequency-domain response, with vibration energy shifting from low frequencies toward mid–high frequencies as narrowing increased. In the clinical cohort, representative cases of significant stenosis and dissection exhibited similar changes in spectral content, motivating the design of compact, interpretable biomarkers capturing high-frequency fraction, spectral shape, and texture-like energy patterns. With grouped cross-validation by patient and side, multivariate models based on six non-redundant features achieved strong discrimination at both the segment level and the clinically relevant patient-side level, and SHAP analysis highlighted which biomarkers most strongly contributed to model outputs. The best-performing model, L2-regularized logistic regression, achieved AUROC 0.970 and AUPR 0.947 with accuracy 0.898 (sensitivity 0.817, specificity 0.936, precision 0.854, F1 0.835), supporting vibration-based carotid sensing as a feasible and physiologically grounded pathway toward accessible, scalable screening and longitudinal monitoring of carotid artery disease and motivating external validation.

## Figures and Tables

**Table 1:** Segment-level test performance across classifiers using the selected six time–frequency biomarkers. Metrics are reported on the held-out test set at the prespecified operating threshold.

Model	AUROC	AUPR	Sensitivity	Specificity	Precision	F1	Accuracy
Logistic Regression	0.970	0.947	0.817	0.936	0.854	0.835	0.898
Random Forest	0.959	0.926	0.804	0.899	0.786	0.795	0.869
ExtraTrees	0.957	0.922	0.834	0.877	0.758	0.794	0.864
XGBoost	0.956	0.922	0.847	0.887	0.775	0.810	0.874

## Data Availability

The data presented in this study are available on request from the corresponding authors.

## Supplementary Materials

The following supporting information can be downloaded at https://www.mdpi.com/article/10.3390/bios16050238/s1, Figure S1: Seismometer patch placed on each carotid artery on each side of the neck. (a) Example of MEMS die. (b) OPS MEMS die picture taken with a scanning electron microscope (SEM). (c) IPS MEMS die picture taken with a scanning electron microscope (SEM). (d) Allan deviation plot of both OPS and IPS exhibiting low-noise performance of 6.7 µg/√Hz and 8.6 µg/√Hz, respectively; Figure S2: (a) Vessel phantom: A 5-mm silicone tube embedded in Humimic Gel #1 with a localized constriction; seismic patch adhered over the vessel and secured with medical tape. (b) Phantom arteries: Constricted (left) and healthy (right). (c) Closed-loop system diagram: pump, blood-mimicking fluid, and phantom vessel. Constriction of (d) 0%, (b) 25%, (c) 50%, (d) 75%. Continuous wavelet transform (analysis band 5–60 Hz); color denotes normalized wavelet power (arbitrary units). Increasing constriction shifts energy from <20 Hz to higher frequencies and broadens the spectrum, consistent with the transition from near-laminar to turbulent flow; Figure S3: Univariate discriminative ranking of selected time–frequency biomarkers on the training set. Bars show the mean rank across AUROC and AUPR (lower is better) computed with patient-side grouped cross-validation; Figure S4: Representative analytic Morse (3, 60) wavelet from the CWT filter bank, shown here at a center frequency of 30.4 Hz within the 5–60 Hz analysis band used in this study; Table S1: Participant demographics and side-specific carotid condition labels for the clinical cohort. Age, height (m), and body mass index (BMI; kg/m^2^) are reported for each participant, along with the corresponding left and right carotid clinical status; Table S2: Univariate performance (AUROC/AUPR; 5-fold patient-side grouped CV on training data) and Mann–Whitney U test results (BH-FDR; patient-side aggregated) for QC-passed features, with Cliff’s delta and direction; Table S3: Patient-side test-set performance across classifiers using the selected six time–frequency biomarkers. Segment probabilities were aggregated within each patient side by averaging to produce one probability per carotid side. The operating threshold was selected from training predictions and applied unchanged to the held-out test set. Metrics reported include AUROC, AUPR, sensitivity, specificity, precision, F1, and accuracy; Table S4: Demographic ablation results using logistic regression with patient-side grouped 5-fold cross-validation (segment probabilities averaged per patient-side). Performance (mean ± SD) is reported for signal-only (Base6), demographics-only (age, sex, height, weight, BMI), and signal + demographics feature sets; Table S5: Leave-one-feature-out ablation of the final six-feature logistic regression model. Grouped patient-side cross-validated performance on the training set.
