# Supplementary material for: Automated Detection of Carotid Artery Stenosis Using a Sensitive Accelerometer Wearable Sensor and Interpretable Machine Learning
Source: Biosensors (Basel). 2026 Apr 23;16(5):238. doi: 10.3390/bios16050238 (PMC13204257; doi:10.3390/bios16050238)
Supplement: Supplementary file 1 [file biosensors-16-00238-s001.zip › biosensors-4194088-supplementary.pdf]

Supplementary Materials

Seismometer Patch Specifications

We used a hermetically sealed microelectromechanical system (MEMS) accelerometer with exceptionally high out-of-plane sensitivity, achieving micro-g/√Hz noise performance across a wide ±6 g dynamic range and a 10 kHz bandwidth (Figure 1a). The device operates both as a conventional DC accelerometer and as a wideband vibrometer (contact microphone), allowing it to capture low-frequency chest motion from breathing as well as higher-frequency chest vibrations associated with PIVs.

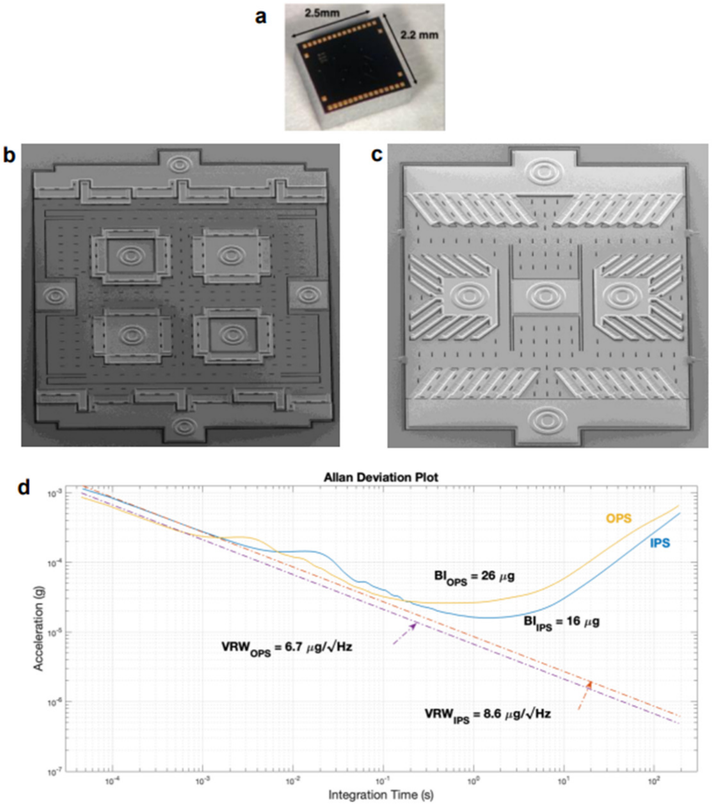

Figure S1. Seismometer patch placed on each carotid artery on each side of the neck. a) Example of MEMS die. b) OPS MEMS die picture taken with a scanning electron microscope (SEM). c) IPS MEMS die picture taken with a scanning electron microscope (SEM). d) Allan deviation plot of both OPS and IPS exhibiting low-noise performance of 6.7 μg/√Hz and 8.6 μg/√Hz, respectively

Supplementary Table S1. Participant demographics and side-specific carotid condition labels for the clinical cohort. Age, height (m), and body mass index (BMI; kg/m²) are reported for each participant, along with the corresponding left and right carotid clinical status

Patient #	Age	Height (m)	BMI	Gender	Weight(kg)	Condition (Left / Right)
1	69	1.676	27.13	F	76.2	Left: Stenosis; Right: No stenosis
2	62	1.829	27.8	M	93	Left: No stenosis; Right: No stenosis
3	61	1.692	38.77	F	111	Left: No stenosis; Right: No stenosis
4	46	1.651	30.19	F	82.3	Left: Dissection; Right: No stenosis
5	47	1.702	23.34	F	67.6	Left: <50%; Right: <50%
6	60	1.727	29.54	M	88.1	Left: <50%; Right: <50%
7	41	1.575	34.02	F	84.4	Left: <50%; Right: <50%
8	57	1.778	26.26	M	83	Removed due to swallow during recording
9	49	1.6	33.13	F	84.8	Removed due to coughing
10	37	1.6	31.68	F	81.1	Left: <50%; Right: <50%
11	49	1.702	34.38	M	99.6	Left: <50%; Right: >50%
12	52	1.778	41.44	M	131	Left: <50%; Right: <50%
13	49	1.702	18.78	F	54.4	Left: Dissection; Right: FMD
14	46	1.702	22.71	M	65.8	Left, Right: Stenosis, intracranial arteries narrowed
15	69	1.803	32.3	F	105	Removed swallowed during recording
16	42	1.829	34.08	M	114	Left: <50%; Right: <50%
17	81	1.727	25.52	M	76.1	Left: stenosis intracranial Left carotid; Right: <50%
18	54	1.778	21.26	F	67.2	Left: <50%; Right: <50%
19	61	1.6	30.23	F	77.4	Left: <50%; Right: <50%
20	55	1.778	25.24	M	79.8	Left: <50%; Right: <50%
21	74	1.575	28.34	F	70.3	Left: <50%; Right: <50%
22	81	1.499	20.65	F	46.4	Left: <50%; Right: <50%
23	59	1.753	28.05	M	86.2	Left: <50%; Right: <50%
24	71	1.575	26.69	F	66.2	Left: <50%; Right: <50%
25	74	1.6	23.05	F	59	Left: <50%; Right: <50%
26	55	1.638	21.8	F	58.5	Left: <50%; Right: <50%
27	40	1.803	27.5	M	89.4	Left: <50%; Right: <50%
28	73	1.651	27.33	F	74.5	Left: <50%; Right: <50%
29	43	1.854	27.26	F	93.7	Left: Dissection; Right: No stenosis
30	79	1.626	23.19	F	61.3	Left: <50%; Right: <50%
31	72	2	30.15	F	74.8	Left: <50%; Right: <50%
32	60	1.676	30.79	M	86.5	Left: <50%; Right: <50%
33	53	1.676	20.36	M	57.2	Left: Dissection; Right: Dissection
34	74	1.803	25.41	M	82.6	Left: Stenosis>50%; Right: <50%
35	64	1.727	26.02	F	77.6	Left: <50%; Right: <50%
36	76	2	21.75	F	63	Left: Stenosis>50%; Right: <50%
37	67	1.727	23.87	M	71.2	Left: Stenosis >50% ; Right: <50%
38	46	1.829	20.21	F	67.6	Left: <50%; Right: <50%
39	76	99.3	missing	F	missing	Left: <50%; Right: <50%
40	73	1.702	22.23	M	64.4	Left: <50%; Right: <50%
41	63	1.626	31.05	M	82.1	Left: <50%; Right: <50%
42	53	1.778	28.85	M	91.2	Left: <50%; Right: <50%
43	79	1.905	23.12	M	83.9	Left: <50%; Right: <50%
44	78	1.827	22.89	M	76.4	Left: <50%; Right: <50%
45	73	1.829	29.83	M	99.8	Left: Stenosis>50%; Right: Stenosis>50%
46	71	1.575	23.54	F	58.4	Left: <50%; Right: <50%
47	79	1.676	22.14	M	62.2	Left: Stenosis>50%; Right: Stenosis>50%
48	74	1.575	24.31	F	60.3	Left: <50%;; Right: Stenosis>50%
49	83	1.727	22.06	M	65.8	Left: <50%;; Right: Stenosis>50%
50	45	1.676	43.43	F	122	Left: <50%; Right: <50%
51	74	1.651	22.2	F	60.5	Left: <50%; Right: Stenosis>50%
52	62	1.829	24.39	M	81.6	Left: Stenosis>50%; Right: Dissection (pseudoaneurysm)
53	83	1.702	28.17	F	81.6	Left: No stenosis; Right: Stenosis
54	72	1.6	32.19	F	82.4	Left: No stenosis; Right: No stenosis
55	58	1.582	29.01	F	72.6	Left, Right: <50% (intracranial narrowing bilateral carotids)
56	54	1.575	34.47	F	85.5	Left and Right: FMD, <50%, beading
57	74	1.727	28.9	M	86.2	Left and Right <50%
58	73	1.753	27.17	M	83.5	Left and Right <50%
59	42	missing	28.24	F	83	Left and Right <50% stenosis, Right showed dissection
60	42	1.803	22.3	M	72.5	Left: Stenosis> 50%; Right: <50%
61	76	1.715	27.91	F	82.1	Left: Stenosis> 50%; Right: <50%
62	65	1.6	24.06	F	61.6	Left: Stenosis>50%; Right: Stenosis>50%
63	80	1.575	26.32	F	65.3	Left: Stenosis>50%; Right: stenosis<50%
64	85	1.778	23.82	M	75.3	Left: Stenosis>50%; Right: stenosis<50%
65	43	1.702	25.06	M	72.6	Left: stenosis<50% ; Right: <50%stenosis
66	49	1.626	28.82	F	76.2	Left and Right <50%
67	48	1.626	22.01	F	58.2	Left: <50%; Right: <50%
68	78	1.6	23.24	F	59.5	Left: Stenosis>50%; Right: Stenosis>50%
69	80	1.803	33.53	M	109	Left: stenosis<50%; Right: Stenosis>50%;
70	75	1.626	25.08	F	66.3	Left: Stenosis>50%; Right: Stenosis>50%
71	72	1.7526	38.39	M	117.934	Left: stenosis<50; Right: Stenosis>50%
72	75	1.524	18.94	F	44	Left: stenosis<50; Right: Stenosis>50%
73	41	1.575	28.18	F	69.9	Left: stenosis>50; Right: Stenosis>50%
74	83	1.778	23.31	M	73.7	Left: <50%; Right: >50%

Phantom study

We built a benchtop carotid phantom to evaluate whether a wearable seismic patch can non-invasively sense flow disturbances produced by stenosis. A 5-mm-ID silicone vessel was embedded in Humimic Gel #1 to approximate the viscoelastic loading of superficial neck tissue. A commercial blood-mimicking fluid was circulated in a closed loop with a peristaltic pump to generate repeatable pulsatile flow (Fig. 1c). Progressive narrowing of the lumen was emulated with interchangeable in-line restrictors installed in the tubing to achieve nominal stenosis levels of 0%, 25%, 50%, and 75%. The healthy reference lumen was 5 mm; restrictors with inner diameters of 3 mm and 1.6 mm were used to represent moderate and severe narrowing, respectively (about 64% and about 90% area reduction relative to 5 mm). An adhesive StethX wearable seismic patch was placed on the gel surface adjacent to the constriction region and secured with 3M Medipore tape to ensure consistent coupling (Fig. 1a). Photographs of healthy versus constricted segments and a system-level schematic are shown in Fig.

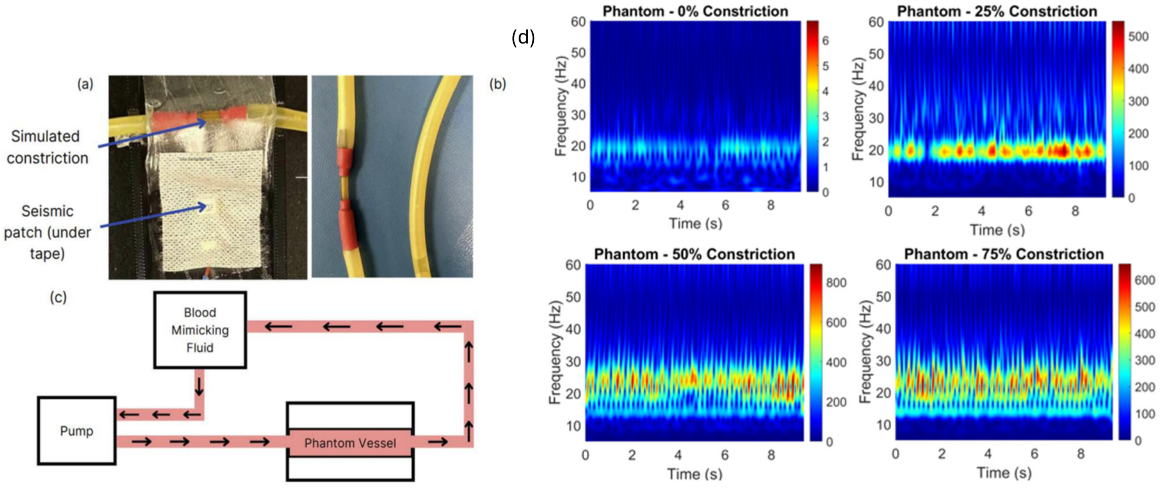

Figure S2. (a) Vessel phantom: A 5-mm silicone tube embedded in Humimic Gel #1 with a localized constriction; seismic patch adhered over the vessel and secured with medical tape. (b) Phantom arteries: Constricted (left) and healthy (right). (c) Closed-loop system diagram: pump, blood-mimicking fluid, and phantom vessel. Constriction of (d) 0%, (b) 25%, (c) 50%, (d) 75%. Continuous wavelet transform (analysis band 5–60 Hz); color denotes normalized wavelet power (arbitrary units). Increasing constriction shifts energy from <20 Hz to higher frequencies and broadens the spectrum, consistent with the transition from near-laminar to turbulent flow.

Graded constrictions in the benchtop phantom produced systematic changes in the time–frequency signatures measured by the wearable patch (Fig. 1d). With 0% constriction, energy was concentrated <~20 Hz and organized into two cardiac-phase ridges, consistent with near-laminar flow. At 25%, a modest rise of power appeared in the 20–30 Hz band. At 50%, power broadened and extended further into the 30–40 Hz range. At 75%, the scalogram exhibited broadband elevation up to ~60 Hz with dense, high-frequency texture, indicating markedly disturbed/turbulent flow.

Analysis:

For descriptive purposes, we also compared the per-carotid distributions of the selected features between stenotic and non-stenotic groups using the Mann–Whitney U test on the training set, with Benjamini–Hochberg false-discovery rate (FDR) correction.

Supplementary Table S2. Univariate feature screening and statistical consistency check. All non-redundant engineered features that passed QC are listed. Univariate discrimination was assessed on the training set using 5-fold stratified grouped cross-validation (group = patient-side) and summarized by AUROC and AUPR; features are ordered by the mean rank of AUROC and AUPR (lower is better). Distribution differences between stenosis and non-stenosis were assessed using a two-sided Mann–Whitney U test with Benjamini–Hochberg false-discovery rate (FDR) correction on patient-side aggregated values; Cliff's delta is reported as a nonparametric effect size.

Supplementary Table S2. Univariate performance (AUROC/AUPR; 5-fold patient-side grouped CV on training data) and Mann–Whitney U test results (BH-FDR; patient-side aggregated) for QC-passed features, with Cliff's delta and direction.

Feature	AUROC	AUPR	Mean rank	Mann Whitney	BH false discovery rate (FDR) q	Cliff delta	Direction
HFPR_top50	0.99	0.985	1	8.73E-16	9.31E-15	0.984	higher_in_stenosis
energy_centroid	0.961	0.943	2.5	1.96E-14	8.96E-14	-0.936	lower_in_stenosis
energy_spread	0.973	0.92	2.5	6.82E-15	3.64E-14	0.952	higher_in_stenosis
SOBEL_full	0.877	0.806	5	7.89E-11	1.94E-10	0.795	higher_in_stenosis
ENT_top_over_bottom50	0.885	0.778	5.5	5.65E-10	1.29E-09	0.758	higher_in_stenosis
RIDGE_row_std	0.912	0.705	5.5	6.85E-13	1.99E-12	0.878	higher_in_stenosis
ENT_top50	0.861	0.791	6	2.49E-08	5.30E-08	0.682	higher_in_stenosis
SPARSITY_gini	0.784	0.666	8.5	3.62E-06	6.09E-06	-0.567	lower_in_stenosis
ENT_renyi2_full	0.781	0.654	10.5	4.84E-06	7.37E-06	0.559	higher_in_stenosis
RIDGE_curvature_l2	0.809	0.571	11	7.89E-11	1.94E-10	0.795	higher_in_stenosis
ENT_full	0.766	0.662	11	7.31E-06	1.06E-05	0.549	higher_in_stenosis
SVD_s1_over_sum	0.774	0.584	12	4.12E-06	6.59E-06	-0.563	lower_in_stenosis
SPARSITY_ipr	0.753	0.664	12	1.28E-05	1.78E-05	0.534	higher_in_stenosis
ORIENT_coherence	0.765	0.629	12.5	2.97E-06	5.28E-06	0.572	higher_in_stenosis
RIDGE_row_mean	0.754	0.555	15	2.79E-07	5.58E-07	-0.628	lower_in_stenosis
TIME_energy_cv	0.697	0.564	15.5	0.005	0.0059	0.344	higher_in_stenosis
SVD_effective_rank	0.696	0.521	17	0.0025	0.0031	0.369	higher_in_stenosis
rolloff85	0.662	0.503	18	0.0019	0.0024	-0.38	lower_in_stenosis
rolloff95	0.558	0.386	19.5	0.4391	0.4532	-0.095	lower_in_stenosis
EDGE_density_p90	0.585	0.324	20	2.52E-06	4.75E-06	-0.576	lower_in_stenosis
RIDGE_slope	0.512	0.346	21	0.8797	0.8796	-0.019	lower_in_stenosis
ORIENT_theta_deg	0.546	0.273	22	0.321	0.3424	0.122	higher_in_stenosis
TIME_LR_balance	0.507	0.288	22.5	0.0844	0.0964	0.211	higher_in_stenosis

Supplementary Table S2 reports univariate screening results for all non-redundant engineered features that passed quality-control checks. Features were evaluated individually on the training set using 5-fold stratified grouped cross-validation (group = patient-side), and discrimination was summarized using AUROC and AUPR computed on held-out folds; features are ordered by the mean rank of AUROC and AUPR (lower is better). As a secondary consistency check, class-wise distribution differences were assessed using a two-sided Mann–Whitney U test on patient-side aggregated feature values with Benjamini–Hochberg false-discovery rate (FDR) correction; Cliff's delta is reported as a nonparametric effect size.

Univariate discriminative ranking of time–frequency biomarkers

We performed univariate screening to quantify the discriminative ability of each engineered time–frequency biomarker in isolation. All univariate metrics were computed on the training set only using patient-side grouped stratified 5-fold cross-validation (grouped by patient-side) to prevent leakage across

segments from the same carotid. Within each fold, the raw feature value was used as a scalar decision score and AUROC and AUPR were computed on the held-out fold; missing values were imputed using the median estimated from the training portion of the fold. To account for unknown score directionality, AUROC and AUPR were computed for both the feature and its negation, and for each metric we retained the orientation with higher cross-validated performance after averaging across folds. Features were then ranked by the mean of their AUROC and AUPR ranks.

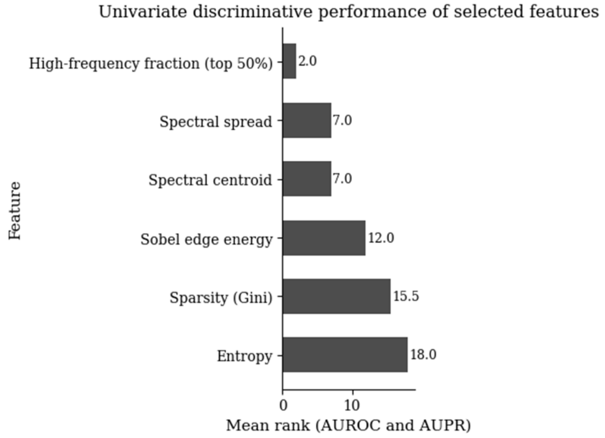

Figure S3. Univariate discriminative ranking of selected time–frequency biomarkers on the training set. Bars show the mean rank across AUROC and AUPR (lower is better) computed with patient-side grouped cross-validation.

Patient-side evaluation

Patient-side evaluation was used as the primary clinical decision unit by aggregating segment-level probabilities within each carotid side. As shown in Supplementary Table S3, all evaluated models achieved similarly strong patient-side discrimination on the held-out test set, indicating that performance was not driven by a specific classifier choice. Thresholded metrics were computed using a prespecified operating threshold selected on the training set and applied unchanged to the test set.

Supplementary Table S3. Patient-side test-set performance across classifiers using the selected six time–frequency biomarkers. Segment probabilities were aggregated within each patient side by averaging to produce one probability per carotid side. The operating threshold was selected from training predictions and applied unchanged to the held-out test set. Metrics reported include AUROC, AUPR, sensitivity, specificity, precision, F1, and accuracy.

Model	AUROC	AUPR	Sensitivity	Specificity	Precision	F1	Accuracy
Logistic Regression	0.988	0.976	0.833	1	1	0.909	0.95
Random Forest	0.988	0.976	0.833	0.929	0.833	0.833	0.9
Extratrees	0.988	0.976	0.833	0.929	0.833	0.833	0.9
XGBoost	0.988	0.976	0.833	0.929	0.833	0.833	0.9

Demographic ablation.

To assess potential demographic confounding, we trained logistic regression classifiers using first the six preselected vibration-derived features as Base6, second, demographics only (age, sex, height, weight, BMI), and third Base6 plus demographics. All experiments used patient-side grouped 5-fold cross-validation to prevent segment-level leakage; segment probabilities were averaged within each patient-side for evaluation (Table S4). Demographics alone provided limited discrimination (AUROC 0.604;

AUPR 0.461), whereas Base6 achieved high performance (AUROC 0.987; AUPR 0.968). Adding demographics did not improve performance (AUROC 0.964; AUPR 0.902), indicating that the model's discrimination is primarily driven by the vibration-derived features rather than demographic variables. We note that weight and BMI contained missing entries, which were treated as missing values and median-imputed.

Supplementary Table S4. Demographic ablation results using logistic regression with patient-side grouped 5-fold cross-validation (segment probabilities averaged per patient-side). Performance (mean ± SD) is reported for signal-only (Base6), demographics-only (age, sex, height, weight, BMI), and signal + demographics feature sets.

Model	Feature set	# features	AUROC (mean ± SD)	AUPR (mean ± SD)
Signal-only (Base6)	HFPR_top50, energy_centroid, energy_spread, Entropy_top50, Sparsity_gini, Sobel_full	6	0.9868 ± 0.0191	0.9675 ± 0.0513
Demographics-only	Age, Sex, Height, Weight, BMI	5	0.6042 ± 0.0542	0.4606 ± 0.0682
Signal + demographics	Base6 + Age, Sex, Height, Weight, BMI	11	0.9643 ± 0.0492	0.9018 ± 0.1424

Leave-one-feature-out ablation. To further characterize the contribution of the selected biomarkers, we performed leave-one-feature-out ablation of the final six-feature logistic-regression model on the training set using patient-side grouped 5-fold cross-validation. Each row in Supplementary Table S5 reports performance after removing one feature from the Base6 set. This analysis was used to assess relative feature contribution within the final compact feature set and was not used to redefine the held-out test model.

Supplementary Table S5. Leave-one-feature-out ablation of the final six-feature logistic regression model. Grouped patient-side cross-validated performance on the training set.

Removed feature	Grouped CV AUROC	Grouped CV AUPR
None (using base6)	0.9844	0.9515
Energy centroid	0.9827	0.9494
Energy spread	0.9848	0.9523
Sobel edge energy	0.9844	0.9516
Sparsity gini	0.9856	0.9547
Entropy	0.9848	0.9500
High frequency fraction top 50%	0.9811	0.9537

Representative wavelet from the CWT filter bank

Figure S4. Representative analytic Morse wavelet used in the CWT analysis. A representative analytic Morse (3,60) wavelet from the CWT filter bank is shown for a center frequency of 30.4 Hz, within the 5–60 Hz analysis band used in this study. Because the CWT is formed from a family of scaled wavelets rather than a single fixed waveform, this panel is provided as an illustrative example of the wavelet shape used in the time–frequency analysis.

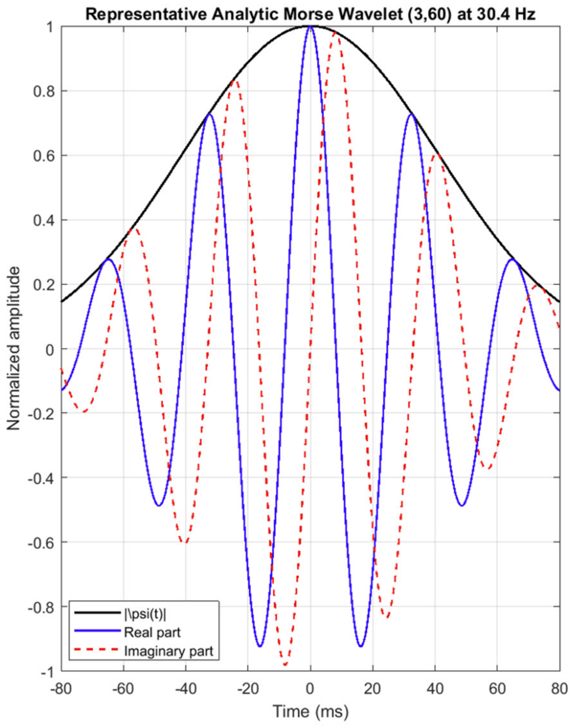

Figure S4. Representative analytic Morse (3,60) wavelet from the CWT filter bank, shown here at a center frequency of 30.4 Hz within the 5–60 Hz analysis band used in this study.
